# Recent Advances in Protein Kinase Activity Analysis Based on Nanomaterials

**DOI:** 10.3390/ijms20061440

**Published:** 2019-03-21

**Authors:** Zhiyong Yan, Pingye Deng, Yang Liu

**Affiliations:** 1 Department of Chemistry, Beijing Key Laboratory for Analytical Methods and Instrumentation, Key Lab of Bioorganic Phosphorus Chemistry and Chemical Biology of Ministry of Education, Tsinghua University, Beijing 100084, China; yanzhiyong@beijinglab.com.cn; 2 Beijing Center for Physical and Chemical Analysis, Beijing 100089, China; dengpy99@tsinghua.org.cn

**Keywords:** nanomaterials, protein kinase, phosphate recognition, signal amplification, signal transduction, biosensors

## Abstract

Protein phosphorylation regulated by protein kinases, as well as their dephosphorylation, is one of the most common post-translational modifications, and plays important roles in physiological activities, such as intracellular signal communications, gene transcription, cell proliferation and apoptosis. Over-expression of protein kinases is closely associated with various diseases. Consequently, accurate detection of protein kinases activities and their relevant inhibitors screening is critically important, not only to the biochemical research, but also to the clinical diagnosis and therapy. Nanomaterials, taking advantage of large surface areas, as well as excellent electrical, catalytic, magnetic and optical properties, have been utilized as target concentrators, recognition components, signal transducer or amplification elements in protein kinase related assays. This review summarizes the recent representative works to highlight the applications of nanomaterials in different biosensor technologies for protein kinases activities detection and their inhibitors screening. First, different nanomaterials developed for phosphoprotein/phosphopeptide enrichment and phosphate recognition are introduced. Next, representative works are selected that mainly focus on the utilization of nanomaterials as signal transducer or amplification elements in various protein kinases sensing platforms, such as electrochemical, colorimetric, fluorescent, and mass spectroscopy-based approaches. Finally, the major challenges and perspectives of nanomaterials being applied in protein kinases related assays are discussed.

## 1. Introduction

Post-translational modifications, such as phosphorylation, glycosylation, methylation, lipidation, ubiquitination, acylation and nitrosylation, are well-known to play fundamental roles in regulation of protein binding affinity, activity and stability [1]. Thus, post-translational modifications are critical in various cellular and extracellular physiological activities. Protein phosphorylation regulated by protein kinases is one of the most common post-translational modifications in the eukaryotic cell and it plays important roles in intracellular signal communications, gene transcription, cell proliferation, apoptosis, etc. [2]. The phosphorylation mechanism of protein phosphorylation has been emphasized by Edmond H. Fischer and Edwin G. Krebs, who were awarded the Nobel Prize in Physiology or Medicine in 1992, “for their discoveries concerning reversible protein phosphorylation as a biological regulatory mechanism”. In principle, protein phosphorylation is accomplished by protein kinases, which catalyze the phosphorylation reactions by transferring the gamma phosphate of ATP, in exceptional cases GTP, to hydroxyl groups of the serine, threonine or tyrosine on protein substrates [3]. The dephosphorylation process is accomplished by the phosphatase. In addition, protein kinases have also been involved in essential non-catalytic functions such as survival, metabolism, differentiation, etc., which were summarized by Rauch et al. [4]. Pharmacological and pathological evidence has proved that the aberrant activities of protein kinases are associated with many human diseases ranging from cancer to Alzheimer’s disease, inflammation, diabetes, cardiovascular diseases, central nervous system disorder, and so on [5,6,7]. Therefore, protein kinases have become a class of drug targets in pharmaceutical industry [8]. The human genome consists of 518 protein kinases genes, and over 150 are proved misregulated or mutated in various diseases. About 80 out of the 518 protein kinases in the human kinome have been targeted [9]. Protein kinase inhibitors have been investigated as the therapeutic reagents against diseases because of their ability to regulate down the activity of protein kinase. The USA Food and Drug Administration (FDA) has approved 31 small molecule kinase inhibitors for human use until November 2016, while many others are currently evaluated in clinical and preclinical trials [10]. Up to now, protein kinase inhibitors have been applied to drug design [11], cancer treatment [12], herpesvirus-associated disease [13], inflammatory and autoimmune out disorders [14], and so on. Consequently, protein kinase activity profiling and relevant inhibitor screening are critically important, not only to the biochemical research, but also to the diagnosis and therapy.

The past few decades have witnessed significant progress in protein kinase related assays. Up to now, various methods have been reported for protein kinase activity analysis and inhibitor screening, such as radiometric, colorimetric, electrochemical, fluorescent approaches, etc. Recently, with the development of nanotechnology, new exciting achievements in designing protein kinase biosensors with superior accuracy, specificity and sensitivity have been attained attributing to different kinds of nanomaterials. Due to their specific physical structures, and outstanding optical, electrical, catalytic and magnetic properties [15,16], nanomaterials demonstrate their advantages in phosphoprotein/phosphopeptide enrichment and phosphate recognition fields, as well as signal generation, amplification or transduction mechanisms.

## 2. Phosphoprotein/phosphopeptide Enrichment or Phosphate Recognition by Nanomaterials

Selective enrichment of phosphoprotein/phosphopetide or phosphate recognition is the major challenge to successful assessing of protein kinase activities. Liu et al. summarized the basic phosphor-recognition mechanisms when designing the protein kinase detection strategies [17], such as phosphor-specific recognition protein, metal chelation, ATP analogs labeling, quantification of phosphorylation products, and so on. Among these, phosphor-recognition via metal chelation (e.g., Zr^4+^, Zn^2+^, Ti^4+^, and Cu^2+^) is the most popular method. Another common method is based on the phosphor-specific recognition protein, such as antigen–antibody interaction or biotin-avidin bridge. Moreover, the quantification of protein kinase can also be performed by directly measuring the phosphorylation with the help of mass spectrometry (MS) [18]. However, these methods are limited by several factors, such as the recognition efficiency, the stability and economy in complicated bio-samples. Recently, benefitting from the development of nanotechnology, different kinds of functional nanomaterials for efficiently phosphor-specific recognition have been developed based on several enrichment principles, such as electronic attraction, chelating and noncovalent interactions [19].

### 2.1. Metal Oxides and Metal Oxides Composites

Many transition metal oxides are capable of interacting strongly with small phosph(on)ates. Besides, it has been proved that the alkyl phosphates self-assemble on the surface of metal oxide nanoparticles such as SnO_2_, TiO_2_, Al_3_O_4_, ZrO_2_, and ZnO. Therefore, these metal oxide nanoparticles can be utilized for phosphopeptide enrichment [20,21,22]. For instance, TiO_2_ combined with specific modified imprinted polymer was designed and provided more than 90% selectivity for phosphopeptides [23]. A ZnO nanorods array-based field-effect transistor biosensor was developed for phosphate detection [24]. In addition, the relevant composites of the metal oxide can also be utilized for the enrichment of phosphorylated proteins/peptides [25,26]. For example, Li and co-workers designed and synthesized SnO_2_-ZnSn(OH)_6_, which was later applied as efficient probes for the phosphopeptide enrichment [27].

### 2.2. Magnetic Nanomaterials

To target phosphoprotein/phosphopeptide rapidly and efficiently, superparamagnetic nanomaterials combined with the fundamentals of metal ions affinity in enrichment of phosphoprotein/phosphopeptide have been the focus of recent studies [28]. These modified magnetic nanomaterials can be manipulated by an external magnetic field, thus they provide a harmless and simple way to enrich and separate phosphoprotein/phosphopeptides from complex samples. For instance, Ta_2_O_5_-coated magnetic nanoparticles (Fe_3_O_4_@Ta_2_O_5_), TiO_2_-coated magnetic nanoparticles (Fe_3_O_4_@TiO_2_) and ZrO_2_-coated magnetic nanoparticles (Fe_3_O_4_@ZrO_2_) can act as affinity probes for selective enrichment of phosphopeptides, and Fe_3_O_4_@Ta_2_O_5_ show better sensitivity and selectivity than Fe_3_O_4_@TiO_2_ and Fe_3_O_4_@ZrO_2_ [29]. In addition, special-designed magnetic nanocomplex can be designed for multi-phosphorylated species recognition. In a representative example, Fe_3_O_4_@nSiO_2_@mSiO_2_/TiO_2_-Ti^4+^ were synthesized to enrich mono- and multi-phosphorylated phosphopeptides. In this proposal, TiO_2_ was firstly modified on the surface of mesoporous silica, followed by being grafted with 3-(trihydroxysilyl)propyl methylphosphonate. After chelating Ti4^+^ ions, the resulted nanocomplex could successfully enrich seven mono-phosphopeptides and eight multi-phosphopeptides from nonfat milk [30].

### 2.3. Mesoporous Nanomaterials and Porous Metal-Organic Frameworks (MOFs)

Mesoporous nanomaterials, with tridimensional structures, high surface areas and numerous nanoscale pore channels, are widely utilized in phosphopeptides enrichment [31]. For instance, to enrich phosphopeptides from Hela cell extracts, hollow magnetic macro/mesoporous TiO_2_ nanoparticles with large pore volume (0.52 cm^3^ g^−1^) were synthesized, showing better selectivity and sensitivity for phosphopeptide when compared with hollow magnetic mesoporous TiO_2_ with small pore volume (0.37 cm^3^ g^−1^) and solid magnetic macro/mesoporous TiO_2_ [32]. Moreover, metallic zirconia nanoparticles-incorporated ordered mesoporous carbon composites were synthesized and showed excellent hydrophilicity for the selective enrichment of phosphopeptides [33]. However, it is worth noting that mesoporous nanomaterials with deep channel structures may influence the release and enrichment of target.

MOFs are a series of porous materials and they possess designable structures and ultrahigh surface areas. MOFs have attracted broad research interests in many fields such as catalysis, separation, storage, drug delivery, and fuel cell [34,35]. Recently, Functionalized MOFs are investigated in specific enrichment of phosphopeptides [36,37]. For instance, dual-functionalized MOFs, combining the affinity of immobilized metal ion affinity chromatography (IMAC) and metal oxide affinity chromatography (MOAC), were synthesized [38]. The Zr-O cluster acted as MOAC and immobilized Ti^4+^ served as IMAC. It showed high binding capacity and good post-enrichment recovery for phosphopeptide. Moreover, Au and Pt nanoparticles loaded UiO-66 (Au&Pt@UiO-66) nanocomposites were synthesized by our group. [39]. After the kemptide was catalyzed by protein kinase A (PKA), Au&Pt@UiO-66 were chelated to the functionalized electrode by forming Zr-O-P bonds between UiO-66 and the phosphorylated kemptide. The electrogenerated chemiluminescence (ECL) signal of luminol was generated and amplified attributing to the synergistic catalytic activity of Au&Pt@UiO-66 to luminol-H_2_O_2_ reaction, thus an ECL biosensor was fabricated for PKA activity detection and inhibitor screening. In addition, MOFs with magnetic properties have been developed for the enrichment of phosphopeptides [40]. For example, hydrophilic magnetic amino functionalized MOFs (Fe_3_O_4_@PDA@UiO-66-NH_2_) were applied for phosphopeptide enrichment based on the strong binding between Zr and phosphopeptide [41].

### 2.4. Rare Earth-Based Nanomaterials

Recently, Sumaoka and co-workers demonstrated that lanthanide ions and their complexes such as Terbium (III) complexes show high selectivity to phosphotyrosine (pTyr) [42]. Another similar study was reported by Hussain et al., who fabricated diamond-lanthanum oxide and diamond-samarium oxide nanocomposites and applied them to selectively capture phosphorylated peptides from β-casein and BSA. Furthermore, the nanocomposites were utilized to capture the endogenous serum phosphopeptides directly [43].

## 3. Nanomaterials Utilized in Different Biosensor Technologies for Protein Kinase Activity Analysis

During the reaction catalyzed by protein kinase, phosphate groups are transferred from ATP to the substrate peptide/protein. Thus, the common method to introduce detective signal into protein kinase assays are modifying or marking the ATP, substrate peptide/protein or the related biomolecules with electroactive or fluorescent tags. Nanomaterials have shown their promising in generating, transducing or amplifying signals when designing biosensors due to their unique physical, chemical and optical properties. Herein, we summarize the representative works to demonstrate the utilization of nanomaterials in different biosensor technologies for protein kinase activity detection. It is shown that the recognition efficiency and sensitivity of the biosensors can be largely improved, attributing to the advantages of various nanomaterials.

### 3.1. Electrochemical Technique-Based Biosensor for Protein Kinase Activity Analysis

In recent years, considerable research attention has been devoted to integrating electronic elements with recognition and amplification elements to develop electrochemical biosensors. Particularly, nanomaterial-based electrochemical signal transduction and amplification show great promise for improving the sensitivity and selectivity of biosensors. When being applied to construct electrochemical sensing platforms, the nanomaterials not only act as the suitable immobilization matrices, but also bring synergistic effects among catalytic activity, conductivity and their physical structure, which would facilitate the signal transduction as well as signal amplification. In addition, when functionalizing nanomaterials with recognition groups, the recognition and amplification function would integrate and contribute more possibilities when designing electrochemical biosensors for protein kinase activity detection.

#### 3.1.1. Electrochemical Biosensor for Protein Kinase Activity Analysis

Up to now, many electrochemical sensing strategies based on nanomaterials have been developed for the protein kinases activity analysis. For instance, an electrochemical sensing platform for PKA and casein kinase II (CK2) activities detection and inhibition was fabricated by coupling with carboxypeptidase Y (CPY)-assisted peptide cleavage reaction and vertically ordered mesoporous silica films (MSF). When the peptide was phosphorylated in the presence of protein kinase, it resisted CPY cleavage, thus the phosphorylated peptide was adsorbed on the surface of MSFs modified ITO electrode. This hampered the diffusion of electroactive probe FcMeOH to the electrode surface, which led to a detectable reduction of electrochemical signal [44]. Among multitudinous nanomaterials, metal nanoparticles such as gold nanoparticles (AuNPs) and silver nanoparticles (AgNPs) have received significant interest in electrochemical sensing platforms for their excellent conductivity, catalytic activity an special optical properties [45]. For instance, a composite of AuNPs and biotinylated β-galactosidase was utilized in an electrochemical biosensor, and showed excellent sensitivity and selectivity for PKA detection [46]. 

#### 3.1.2. ECL Biosensor for Protein Kinase Activity Analysis

ECL technique is an outstanding tool in terms of sensitivity and signal/noise ratios. ECL is the process in which the redox reaction takes place on the surface of an electrode, accompanied with high energy electron transfer reactions in solution. When the generated excited state molecules return to the ground state, the emission of light is recorded. The ECL biosensor combines the merits of chemiluminescence and electrochemistry and it has been widely utilized in biochemical analysis. Nanomaterial-based ECL biosensors have been the research focus in protein kinase activity detection strategies [47,48]. For instance, An AuNPs mediated dual-potential ECL ratiometric approach was reported for protein kinase activity and inhibition assay. It was based on the simultaneous decrease of cathodic ECL from graphene quantum dots (GQDs) and enhancement of anodic ECL from luminol. AuNPs modified on the phosphorylated peptide through Au-SH bond acted as catalyst to accelerate the oxidation of luminol on the electrode surface. Moreover, the spectra overlap between the absorption band of AuNPs and the ECL emission band of GQDs facilitated the ECL resonance energy transfer between AuNPs and GODs [49]. Our group reported an ECL biosensor for PKA activity detection. The ECL signal was amplified due to the outstanding catalytic activity of AuNPs toward luminol ECL reaction and the large surface areas that carried plenty of xanthine oxidase and DNA [50].

#### 3.1.3. Photoelectrochemical (PEC) Biosensor for Protein Kinase Activity Analysis

PEC technique has aroused special attention in designing electrochemical sensing platforms. In general, light is utilized to excite the PEC active species, and then it is translated into electric energy directly or indirectly; hence, the generated photocurrent can be utilized as the detection signal. With remarkable achievements in nanoscience, more and more photoactive nanomaterials have been investigated and utilized in the PEC sensing platform such as AuNPs [51], quantum dots (QDs) [52], TiO_2_-BiVO_4_ heterostructure [53], MoS_2_/WS_2_ heterojunction [54], and so on. Compared with the traditional photoactive materials such as dyes, they possess better photoelectric conversion efficiency, photostability and biocompatibility. Our group designed a dye-sensitized and localized surface plasmon resonance (LSPR) enhanced PEC biosensor for PKA activity detection. The photocurrent generated by the [Ru(bpy)_3_]^2+^ dyes was amplified by the LSPR of AuNPs when irradiated by visible light. The detection limit of the biosensor was 0.005 UmL^−1^ [55]. Different from the traditional interaction on the surface of an electrode, Ai’s group developed an innovative PEC method for PKA activity detection in solution [25]. In their strategy, graphite-like carbon nitride (g-C_3_N_4_) and TiO_2_ nanocomplex were utilized for conjugating phosphate groups and providing PEC signal, while the signal amplification was triggered by PAMAM dendrimer and alkaline phosphatase; this strategy provided the detection limit of 0.048 UmL^−1^. Table 1 shows recent applications of different nanomaterial-based electrochemical biosensors for protein kinase activity analysis.

### 3.2. Fluorescent Biosensor for Protein Kinase Activity Analysis

Fluorescent technique features high sensitivity, design flexibility, high-throughput capability and automatic operation, and it has recently emerged as a powerful tool in protein kinase related assays [62]. In the traditional fluorescent biosensors with organic fluorescent reagents as the fluorogenic substrates, enzymes are usually utilized to cleave the fluorescent group and the quencher in the organic fluorogenic substrates, followed by generating fluorescent signals. However, these fluorogenic substrates suffer from limited biocompatibility and luminescence intensity. Moreover, they are usually sensitive to photobleaching. Recently, fluorescent metal nanomaterials such as QDs with unique optical properties, have drawn research interests in fluorescent sensors. QDs possess the advantages of wide excitation spectrum, narrow emission spectrum, adjustable emission wavelength, high fluorescence intensity, large stokes shift and light stability, thus they are suitable as fluoresce resonance energy transfer (FRET) donor. For example, Wang et al. developed a single QD-based nanosensor for sensitive detection of cAMP-dependent protein kinase [63]. In their proposal, the phosphorylated and biotinylated peptides were assembled onto the surface of streptavidin-functionalized QDs after catalyzed by protein kinase, then the FRET between QDs and peptide occurred and the signal was recorded. Different from Wang’s work, a FRET biosensor based on Zn^2+^-mediated FRET between QDs and dye-tethered peptides was reported for protein kinase activity analysis by Lim and co-workers [64]. It is interesting that without any surface modification of QDs, Zn^2+^ was the only metal ion that enabled the phosphorylated peptides to attach on the carboxyl groups of the QD surface via metal coordination.

Other than QDs, many other novel nanomaterials are developed as the fluorescent probes. For instance, a double-strand-templated DNA Cu nanoclusters (CuNCs) were utilized in a fluorescent biosensor for PKA activity detection. In the presence of ATP, the CuNCs could not be formed because ATP would combine with its aptamer. However, after catalyzed by PKA, ATP was translated into ADP, thus fluorescent CuNCs were formed, during which the varied fluorescent signal was utilized for PKA activity detection and inhibitor H-89 screening [65]. In addition, Zhang et al. reported the simultaneous determination of PKA and CK2 based on blue-emissive CuNCs and red-emissive AuNCs [66]. In the absence of protein kinase, the peptides capped on the nanoclusters were digested by CPY, resulting in fluorescence quenching of the nanoclusters. In the presence of PKA and CK2, the fluorescence of the nanoclusters was retained because the phosphorylation modification protected the peptides from CPY. Accordingly, the quantitative determination of PKA and CK2 could be achieved simultaneously.

Nanomaterials can also act as fluorescence quenchers. For example, GO is well-known for its efficient quenching effect in fluorescence assays. Huang et al. developed a GO-based multiplex fluorescent sensing platform for PKA, protein kinase Ab1 and protein kinase Src detection. In their proposal, the dye-labeled peptide substrate exhibited strong fluorescence; however, when catalyzed by protein kinase, the phosphorylated peptide carrying both the biotin site and the dye assembled onto streptavidin-coated GO via streptavidin-biotin chemistry, leading to the fluorescence quenching [67]. Wang et al. reported a DNA-hosted CuNCs (dsDNA-CuNCs)/GO-based fluorescent biosensor for PKA detection [68]. The dsDNA-CuNCs were absorbed onto GO surface through π–π stacking interactions. Then, the GO quenched the fluorescence of dsDNA-CuNCs through FRET, thus the PKA could be detected according the change of the fluorescence signal.

### 3.3. Colorimetric Biosensor for Protein Kinase Activity Analysis

Colorimetric biosensors are other common method for protein kinase activity detection. In general, the color intensity of the reaction system will change after the noble metal nanomaterials are aggregated or dispersed, as induced by the analyte. Thus, the manipulation procedure of colorimetric biosensor is easy-to-operate and the result can be easily distinguished by naked eye or simple spectrometry. AuNPs, gold nanorodes (AuNRs) and AgNPs are the main signal indicators for colorimetric-based assays for their high extinction coefficients and LSPR [69,70]. There are two general mechanisms for colorimetric protein kinase biosensors: one is based on the cross-linking aggregation of AuNPs functionalized with recognition elements (e.g., avidin, antibody, ions) or kinase-specific peptide, and the other is based on the non-cross-linking aggregation of unmodified AuNPs, which is tuned by the phosphorylation-induced net charge change of substrate peptide. For instance, a colorimetric protein kinase biosensor based on the phosphorylation-tuned crosslinking of AuNPs was reported [71]. In this strategy, biotinylated peptide combined with streptavidin-functionalized AuNPs through the specific streptavidin-biotin binding and electrostatic interactions, which resulted in the crosslinking and coagulation of AuNPs. However, after the biotin-peptide was phosphorylated by PKA, its net charge altered, which weakened the electrostatic interaction between the phosphopeptide and AuNPs. Therefore, the crosslinking and settlement of AuNPs were prevented. By viewing the color changes of the AuNPs, the PKA activity could be easily detected by the naked eye.

### 3.4. MS-Based Biosensor for Protein Kinase Activity Analysis

As a label-free detection technique, MS is utilized in protein kinase activity analysis and several relevant works have been reviewed. For instance, large-scale profiling of protein kinase, which mainly focuses on peptide array technology or proteomic approaches based on liquid chromatography-MS, has been reported [72]. In addition, MS coupled with affinity enrichment approaches, for the proteome-wide capture, identification and quantification of protein kinase, ATPases, GTPases and other nucleotide-binding proteins have been reviewed [73]. For nanomaterials, they play non-negligible roles in protein kinase detection in several MS-based sensing strategies. For instance, Kim and co-workers developed Ab1 kinase assays on peptide-conjugated AuNPs by using secondary-ion MS imaging, during which the AuNPs served as signal enhancers and target concentrators. In their proposal, AuNPs were first modified with two cysteine-terminated peptides, and then they were blocked by succinimidyl poly(ethylene glycol). After being catalyzed by Ab1 or PKA kinase, a straightforward change in the mass of peptide substrates was generated [74].

### 3.5. Other Biosensor Techniques for Protein Kinase Activity Analysis

The pursuit of sensitive and selective techniques for protein kinase activity analysis has received significant attention. There are many other biosensor technologies adopted for protein kinase detection while nanomaterials play important roles, such as Surface-Enhanced Raman Scattering (SERS), Resonance Light Scattering-based sensing (RLS), and Magnetic Resonance Imaging (MRI). 

SERS-based technique is a useful analytic tool that enhances the Raman scattering signal from molecules in the proximity of metal surface. The charge-transfer process between the analyte and substrate can contribute SERS when it is in resonance with excitation wavelength. With suitable SERS substrate, the SERS based biosensors can identify target in trace concentration; moreover, the sensitivity may achieve single-molecule detection limit. Thus, efficient SERS substrate is highly necessary for the SERS sensing platform. Many kinds of nanomaterials have been investigated as the SERS substrate: AgNPs [75], sulfonated reduced GO/AgNPs composite [76], magnetic nanocomposites [77], etc. Among them, He et al. developed a colloidal SERS-based scheme for PKA activity detection using gold nanostars as a SERS substrate [78].

RLS occurs when the energy of incident beam is close to an absorption band produced by an oscillating dipole. The RLS signals can be amplified and detected by a conventional spectrofluorometer when dipoles are strongly coupled [79]. Due to their strong resonance light scattering, metallic nanoparticles can act as sensitive optical labeling probes for the development of RLS-based sensors: AuNPs [80], QDs [81], magnetic nanoparticles [82], CuS nanosheets [83], etc. For instance, Wang’s group utilized a peptide microarray-based RLS assay with AuNPs as probes for PKA detection in different cell lysates [84]. 

MRI provides a noninvasive method for dynamically sensing physiological events with high spatial and temporal resolution. There are few reports about its usage in protein kinase activity. Shapiro et al. designed a series of protein nanoparticles and then utilized them to construct the iron storage protein ferritin-based sensors; with the help of MRI, the activity of PKA can be detected [85].

## 4. Conclusions and Outlook

The development of a simple, rapid and sensitive sensing platform for protein kinase activity and inhibitor screening is critically important for the signal communication study, clinical early diagnosis of diseases and drug discoveries. Various biosensors based on different techniques have been developed for protein kinase activity detection in the past years, and nanomaterials play vital roles in improving the analytical performance of these biosensing systems. Among these developed biosensing systems, the fluorescent biosensors are supposed to be the most promising approaches for point-of-care (POC) protein kinase activity detection, attributing to their relatively high sensitivity, high-throughput capability, automatic operation and controllable signal readout. The real-time application of other biosensors, such as electrochemical, colorimetric, MS, SERS, RLS and MRI biosensors are usually limited by several disadvantages. For instance, the commonly designed electrochemcal biosensors are usually fabricated on the surface of electrodes, which is complicated and requires repeated incubation, washing and modification steps. Furthermore, the fabricated biosensors are sensitive to their surroundings, such as pH, ion concentration and temperature. The colorimetric biosensors are visualized and convenient; however, the noble nanoparticles are usually unstable. Moreover, the complicated samples will present a non-negligible background signal, thus the sensitivity and accuracy of colorimetric biosensors are greatly influenced. As for MS, SERS, RLS and MRI biosensors, few have been investigated in protein kinase activity study, and they usually need large and expensive instruments to finish the data collection and analysis procedure, which are inconvenient for routine field analysis and POC detection. 

Although exciting progress has been achieved and most biosensors mentioned in this review have been utilized in cell lysates for protein kinase activity detection, very few of them have been tried on real samples. Many factors limit their applications in the real word. For instance, the fabricated biosensors may affect the natural state of cells; the extraneous nanoparticles utilized in biosensors may be unstable or toxic to cells; and the interferences would influence the physical, chemical and optical properties of the nanomaterials, thus the sensitivity and accuracy of the biosensors can be greatly influenced. Moreover, there is a possibility that the reaction mechanism occurred in pure samples and in complicated real samples would be different. Therefore, more stable, specific, selective, and biocompatible nanomaterials toward phosphoprotein/phosphopeptide or phosphate from complex sample matrices such as serum, plasma have to be further expanded, and novel promising nanomaterials as probes to visualize the regulation of protein kinase in their cellular natural environment are needed. Moreover, convenient and efficient separation methods have to be developed to eliminate the influence of interferences. In addition, further fundamental research should be done before and after actual applications. Besides the above-mentioned points, portable, economical, and high-throughput analytical devices are still challenging, because they need multi-disciplinary expert cooperation. 

## Figures and Tables

**Table 1:** Recent typical applications of nanomaterial-based electrochemical biosensors for protein kinase activity analysis.

Nanoparticles	Roles	Phosphate Group Recognition	Targets	Detection Limit	Ref.
AuNPs	Enlarge electrode surface/Carriers/Signal amplification	Specific binding	Protein tyrosine kinase-7	372 fM	[56]
AuNPs	Carriers/catalyst	Zr^4+^ coordination	PKA	0.09 UmL^−1^	[50]
GQDs/Graphene Oxide (GO)	Donor and acceptor	Antibody-antigen interaction	CK2	0.023 UmL^−1^	[57]
Graphite-like C_3_N_4_/AuNPs	Carriers/photoactive materials	Phos-tag-biotin	PKA	0.01502 UmL^−1^	[58]
MOFs	Carriers/surface defect recognition	Zr-O-P bonds	PKA	0.0049 UmL^−1^	[59]
Bi_2_S_3_/AuNPs	Carriers/photoactive materials	Biotinylated Phos-tag	PKA	0.017 UmL^−1^	[60]
phosphorylated-g-C_3_N_4_	Photoactive materials/signal transduction	Zr^4+^ coordination	PKA	0.077 UmL^−1^	[61]
Au and Pt nanoparticles loaded MOFs	Enlarge electrode surface/Catalyst/surface defect recognition	Zr-O-P bonds	PKA	0.009 UmL^−1^	[39]
